# Activating BRAF G469A Missense Mutation in a Pediatric Patient With High-Grade Glioma

**DOI:** 10.1093/jnen/nlab102

**Published:** 2021-12-28

**Authors:** Adam Kronish, Bradley DeNardo, Colin Kanach, Rishi R Lulla

**Affiliations:** 1 Department of Pediatrics, The Warren Alpert School of Medicine at Brown University, Hasbro Children’s Hospital/Rhode Island Hospital, Providence, RI, USA; 2 Department of Pathology, The Warren Alpert School of Medicine at Brown University, Hasbro Children’s Hospital/Rhode Island Hospital, Providence, RI, USA

**FIGURE 1.**
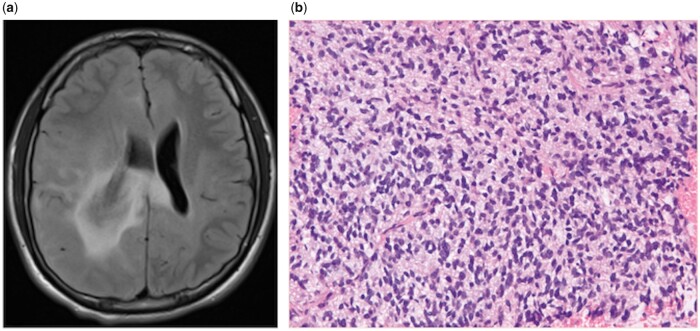
**(A)** Initial diagnostic T2/FLAIR-sequence MRI demonstrating hyperintensity within the right parietal periventricular white matter with involvement of the ipsilateral cortical and subcortical right frontal, temporal, parietal, and occipital lobes with extension across the corpus callosum into the left frontal and parietal lobes. Associated 8 mm of left midline shift at level of 3rd ventricle. **(B)** Microscopic examination with hematoxylin and eosin (H&E) staining of tumor with neuropil background demonstrating hypercellularity, nuclear atypia and scattered mitoses.

To the Editor:

A 15-year-old male presented after a first-time generalized tonic-clonic seizure preceded by headache with left-sided numbness and was found on CT scan to have a right-sided brain mass with midline shift to the left. MRI of the brain further characterized a diffuse lesion centered within the right periventricular parietal white matter with cortical involvement both ipsilaterally and contralaterally consistent with radiologic diagnosis of gliomatosis cerebri (Fig. 1A). The tumor was considered unresectable based on anatomic location and a biopsy was performed. After an uneventful postoperative recovery, the patient was discharged home on systemic glucocorticoids and levetiracetam.

Histologic features of the tumor included hypercellularity with nuclear pleomorphism, rare mitoses, and focal areas of increased anaplasia (Fig. 1B). Immunohistochemical (IHC) stains were positive for GFAP and p53 with negative staining for ATRX, consistent with astrocytic origin. Ki-67 was positive in 10% of cells, and IDH-1 was wild-type by IHC. The final pathologic diagnosis was grade 3 anaplastic astrocytoma. Molecular biologic studies identified partial methylation of the *MGMT* promoter gene and absence of co-deletion of 1p and 19q chromosome arms. An activating mutation of *BRAF* was identified on next-generation sequencing analysis, which was further characterized as a missense mutation: NM_004333.4: c.1406G>C substitution resulting in replacement of glycine at codon 469 with alanine (p.G469A).

The patient was treated with temozolomide and external beam radiation therapy followed by maintenance cycles of lomustine and temozolomide. His treatment course was complicated by breakthrough seizures, behavioral changes, and headache necessitating hospital admissions and modifications of his anti-epileptic regimen. Six months after the completion of radiation therapy, 2 new foci of disease within the radiation field of the right hemisphere were identified on routine surveillance imaging. Given the presence of the activating *BRAF* mutation, he was started on combination BRAF and MEK inhibition with dabrafenib and trametinib. Three weeks after initiation of this therapy, he developed worsening neurologic symptoms and imaging demonstrated further progression of disease with new extensive enhancement of the periventricular and subcortical white matter of the left parietal and temporal lobes. Systemic chemotherapy was discontinued, and the patient’s goals of care were changed to palliation. He entered home hospice care and died within a few weeks.

The BRAF-MEK-MAP kinase pathway is commonly implicated in a variety of human malignancies. This molecular signaling cascade consists of a series of phosphorylation steps starting with Raf proteins and terminating with the phosphorylation of MEK to ERK, thereby mediating gene transcription of proteins essential in the regulation of cell growth and proliferation. Mutations in *BRAF* are one of the most frequent oncogenic molecular events in cancer and in particular have been implicated in malignant melanoma, non-small cell lung cancer, papillary thyroid cancer, and glioma. In the pediatric population, *BRAF* V600E mutations are commonly found in low-grade gliomas (LGGs) and approximately 6–10% of high-grade gliomas (HGGs) (1). Clinical trials utilizing dual BRAF and MEK inhibition for newly diagnosed and relapsed patients with V600-mutant gliomas are currently ongoing.

This case represents the first report of a *BRAF* G469A mutation in HGG. This missense mutation was first described in 2 prior reviews of *BRAF* mutations where it was identified in cases of non-small cell lung cancer and melanoma (2, 3). The AACR Project GENIE international data-sharing consortium identifies *BRAF* G469A in 0.17% of cases, with lung and prostate adenocarcinoma representing the highest prevalence (4). This *BRAF* mutation has shown to result in increased kinase activity with phosphorylation of downstream targets including MEK, suggesting that it is an activating mutation and might confer response to dual BRAF and MEK inhibition (2, 3). This mutation can be further classified as a class II *BRAF* mutation, indicating increased kinase activity but not to the same degree as those resulting from class I activating mutations, such as *BRAF* V600D and V600E mutants (2).

*BRAF* G469A has been previously described in pediatric LGG (5). Hennani et al. identified *BRAF* G469A mutations in 4.5% of cases of pediatric LGG evaluated for alternative genetic abnormalities. None of the 123 pediatric HGGs evaluated in this study were found to have *BRAF* G469A. To the best of our knowledge, our case represents the first report of *BRAF* G469A in malignant glioma and implicates this activating missense mutation in the pathogenesis of HGG.

Given the rarity of the mutation, it is not yet known whether BRAF and MEK inhibition is of clinical benefit in patients harboring this *BRAF* G469A mutation, as seen in our patient. Pediatric LGG is responsive to therapeutic BRAF inhibition in up to 44% of cases, suggesting consideration of this treatment strategy in patients such as ours with HGG harboring alternative activating *BRAF* alterations (6). Evaluation of BRAF and MEK inhibition for rare activating mutations warrants additional research in the future.
